# Nanostructuring Effect of Nano-CeO_2_ Particles Reinforcing Cobalt Matrix during Electrocodeposition Process

**DOI:** 10.3390/nano12172923

**Published:** 2022-08-25

**Authors:** Bogatu Nicoleta, Benea Lidia, Buruiană Daniela-Laura, Bașliu Vasile, Celis Jean-Pierre

**Affiliations:** 1 Faculty of Engineering, Dunarea de Jos University of Galati, 47 Domnească Street, 800008 Galati, RO, Romania; 2 Competences Center, Interfaces-Tribocorrosion-Electrochemical Systems, Dunarea de Jos University of Galati, 47 Domnească Street, 800008 Galati, RO, Romania; 3 Cross-Border Faculty, Dunarea de Jos University of Galati, 47 Domnească Street, 800201 Galati, RO, Romania; 4 Department of Metallurgy and Materials Engineering, Katholieke Universiteit Leuven, Kasteelpark Arenberg 44, B 2450-3001 Leuven, Belgium

**Keywords:** nanostructured layer, nano-CeO_2_ particles, cobalt matrix, X-ray diffraction patterns, wettability

## Abstract

The electrodeposition method was used to obtain nanostructured layers of Co/nano-CeO_2_ on 304L stainless steel, from a cobalt electrolyte in which different concentrations of CeO_2_ nanoparticles (0, 10, 20, and 30 g/L) were dispersed. The electrodeposition was performed at room temperature using three current densities (23, 48, and 72 mA cm^−2^), and the time was kept constant at 90 min. The influence of current densities and nanoparticle concentrations on the characteristics of the obtained nanostructured layers is also discussed. An X-ray diffractometer (XRD) was used to investigate the phase structure and cobalt crystallite size of the nanostructured layers, and a contact angle (sessile drop method) was used to assess the wettability of the electrodeposited layers. The roughness of the surfaces was also studied. The results show that the nanostructured layers became more hydrophilic with increasing nanoparticle concentration and increasing current density. In the case of pure cobalt deposits, an increase in the current density led to an increase in the size of the cobalt crystallites in the electrodeposited layer, while for the Co/nano-CeO_2_ nanostructured layers, the size of the crystallites decreased with increasing current density. This confirms the nanostructuring effect of nano-CeO_2_ electrocodeposited with cobalt.

## 1. Introduction

The progress that has been made in industry and technology today offers us unprecedented opportunities. The ability to make and use matter at the micrometric and nanometric scales are just some of the benefits of the progress that science has made in recent decades. Over the years, researchers have made continuous efforts to explore alternatives to conventional materials and have advanced in terms of material performance, making the development of nanocomposite systems particularly tempting [1,2,3]. Nanocomposites are composites in which one or more phases have nanometric dimensions. It should be noted that materials are considered nano when at least one of the dimensions of the components is at the nanometer scale, with typical dimensions of less than 100 nm. Nanocomposite materials are commonly known as materials consisting of two or more different components with well-defined interfaces. In general, one material forms a continuous matrix, while the other provides curing [4,5,6]. With the rapid development of nanotechnology, a multitude of new materials have been produced for use in domains such as medicine [7], electronics [8], pharmaceuticals [9], the energy industry [10], cosmetics [11], the food industry [12], and the chemical industry [13], as well as for the protection and conservation of the environment [14].

Due to the special properties of nanoscale materials, and the ability of the scientific community to develop and produce new nanostructured materials with the potential to be applied in various domains of activity, their commercial demand has been constantly growing [15,16,17,18]. The European nanomaterials market in 2015 generated revenues of over $2.5 billion and is estimated to reach $9.1 billion in 2022, with an annual growth rate of 20.0% in 2016–2022 [19].

There are currently many methods for obtaining nanocomposites, but one of the most well-known and widely used techniques for obtaining nanocomposites is electrodeposition [20,21,22]. Electroplating is considered one of the remarkable methods of producing nanocomposites due to its many advantages over other methods, such as the simplicity of the method, the use of cheap equipment, its low cost, the uniformity of thickness, its high purity, its lack of shape limitations, the homogeneous particle distribution, the high deposition rate, and the possibility of forming several simple layers, with low costs in many different systems [23,24,25]. The electrochemical codeposition of dispersed phases in the metal matrix to obtain a nanocomposite material involves the dispersion of nanoparticles in an electrolysis bath, where different amounts of nanoparticles are incorporated into the matrix [6].

The success of embedding nanoparticles into the metal matrix depends on a number of factors, such as: the current density, the concentration of nanoparticles, the stirring rate required to maintain a homogenous dispersion, the electrolyte pH, and the electrodeposition time [6,26,27]. Numerous authors [28,29,30,31,32,33] have reported research on nanocomposite coatings formed by the electrochemical codeposition of fine particles in a metal matrix from electrolytic solutions.

It has been found that the introduction of ceramic nanoparticles into a metal matrix leads to an improvement in physical and mechanical properties such as corrosion resistance and wear [34]. There has been a lot of information reported in the literature about nanocomposite layers obtained in a nickel matrix, but very little data published on nanocomposite layers obtained in a cobalt matrix [35].

The types of particles that have so far been deposited in a cobalt matrix to obtain composite or nanocomposite layers are: nanoparticles such as Al_2_O_3_ (30 nm) [36], TiO_2_ (30 nm) [37], CeO_2_ (100 nm) [38], and ZrO_2_ (20 nm) [39], and microparticles such as ZrO_2_ (5 µm) [30], UHMWPE (5 µm) [28,29], CeO_2_, La_2_O_3_ (1 µm) [40], and LaCrO_3_ (1 µm) [41].

The use of cobalt as a matrix for electrodeposition in this paper is of interest due to its special properties, which are useful for the manufacture of medical materials [29]. In biomedical applications, cerium oxide nanoparticles have been investigated for their antibacterial [42] and antioxidant [43,44] properties, their increased tribocorrosion and corrosion resistance [20,21,45], and their toxicological activities [46,47].

The aim of this paper is to prove the nanostructuring effect of nano-CeO_2_ particles embedded into a cobalt matrix during an electrocodeposition process, with a large applied current density domain and nanoparticle concentrations. The crystallite size of the cobalt matrix, the nanostructuring effect of the nano-CeO_2_ particles, and the wettability of the obtained nanostructured layers were investigated.

The novelty of this research work is the demonstration of the nanostructuring effect of CeO_2_ nanoparticles on cobalt crystallites when they are electrodeposited with cobalt and included in the cobalt matrix. The inclusion of nano-CeO_2_ in the cobalt matrix led to a decrease in the size of the cobalt matrix crystallites, which was accentuated by an increase in the current density applied to the electrocrystallization or electrodeposition process.

No studies are available in the literature on the nanostructuring effect of the CeO_2_ nanoparticles included in the cobalt matrix during the electrocodeposition or electrocrystallization process of cobalt. Correlations between nanostructuring and the wettability of the nanostructured layers have also not been reported.

## 2. Materials and Methods

The substrate used for the electrodeposition of the Co/nano-CeO_2_ layers was 304L stainless steel, purchased from Direct Line Inox (Bucharest, Romania) the chemical composition of which was: C, 0.030%; Ni, 8.82%; Mn, 1.02%; Cr, 18.44%; Si, 0.75%; P, 0.045%; and Fe, balanced. The 304L stainless steel samples were received in the form of plates measuring 250 mm × 250 mm × 1.2 mm. They were then cut to the size of 25 mm × 25 mm × 1.2 mm. After cutting, the samples were embedded in epoxy resin to facilitate a constant and well-defined active working surface of 17 mm × 25 mm × 1.2 mm, with the active surface being thus 425 mm^2^.

For the electrochemical deposition, a PGZ 301 electrochemical station (Radiometer Analytical SAS, Villeurbanne, France) was used, which was connected to a laptop with VoltaMaster 4 software (Radiometer Analytical SAS, Villeurbanne, France, version 5.10).

The experimental set-up consisted of a three-electrode electrochemical cell made of glass, including a working electrode (WE), which was 304L stainless steel to provide support for the electrodeposited layers; a reference electrode, which was Ag/AgCl with a saturated KCl solution and a potential of 199 mV vs. NHE; and a counter electrode, which was cobalt. The cobalt plate (99.9% purity) that was used as an anode was purchased from Goodfellow in the form of plates measuring 50 mm × 50 mm × 2 mm. To obtain the pure Co and Co/nano-CeO_2_ layers, the cobalt plating electrolyte had the following composition: 300 g/L of CoSO_4_ × 7H_2_O, 50 g/L of CoCl_2_ × 6H_2_O, 30 g/L of H_3_BO_3_, 1 g/L of C_12_H_25_NaO_4_S (sodium dodecyl sulfate), and distilled water.

Alkali metal sulfates and chlorides were introduced into the cobalt electrolyte, especially to increase the electrical conductivity. In addition to this, some salt additions also increased the cathodic polarization, helping to improve the uniformity of the layers. Boric acid is used quite frequently in electrolytic baths that contain acidic solutions of cobalt salts, helping to maintain a constant pH in the solutions.

The advantage of adding surfactants is their dispersal effect on the particles. Surfactants adsorb particles and favor the distribution of particles, so that the particles are evenly distributed on the surface of the material [25,26,27,28,29,30]. All chemical reagents used for the preparation of the cobalt electrolyte were of analytical purity and were purchased from Merck (Darmstadt, Germany). The physico-chemical parameters of the cobalt plating electrolyte were as follows: the pH, was adjusted to 4.21 ± 0.3 by the dropwise addition of 0.1 N HCl, the electrical conductivity was 36.2 mS/cm, and the salinity was 22.8 ppt. The physico-chemical parameters were determined using a Sension + multiparameter. The deposition of pure cobalt layers and Co/nano-CeO_2_ was achieved potentiostatically at the current densities of 23 mA cm^−2^, 48 mA cm^−2^, and 72 mA cm^−2^. The electrodeposition time was kept constant at 90 min and the stirring rate of the electrolyte was kept at 300 rpm. The degree of CeO_2_ nanoparticles that embedded into the cobalt matrix of nanostructured layers ranged from 0 to 20%.

The volume of the electrolyte in the electrodeposition cell was maintained at 160 mL and the temperature was maintained at 22 °C ± 2 °C for both the electrodeposited systems: the pure cobalt layers and the Co/nano-CeO_2_ nanostructured layers. The nanostructured composite layers were obtained by adding three concentrations of CeO_2_ nanoparticles in the cobalt plating electrolyte: 10 g/L, 20 g/L, and 30 g/L.

A stainless steel support was used to obtain the electrodeposited layers. The following technological flow steps were applied: mechanical cleaning with abrasive paper to remove micron irregularities, washing with distilled water, and chemical degreasing by the immersion of the samples in sodium hydroxide (50 g/L) for 10 min at a temperature of 70 °C in order to remove adherent impurities. After these steps, the samples were washed with distilled water, followed by the immediate introduction of the samples into the deposition electrolyte. All electrodeposition tests were repeated three times to verify the reproducibility of the experimental data.

CeO_2_ nanoparticles (CAS no. 1306-38-3) in the form of nanopowder were used as dispersed phase and were purchased from Sigma-Aldrich (Saint Louis, Missouri, United States). Table 1 summarizes the most important physical and chemical properties of cerium oxide. The manufacturer Sigma-Aldrich does not mention the purity of the CeO_2_ nanopowder.

**Figure 1:**
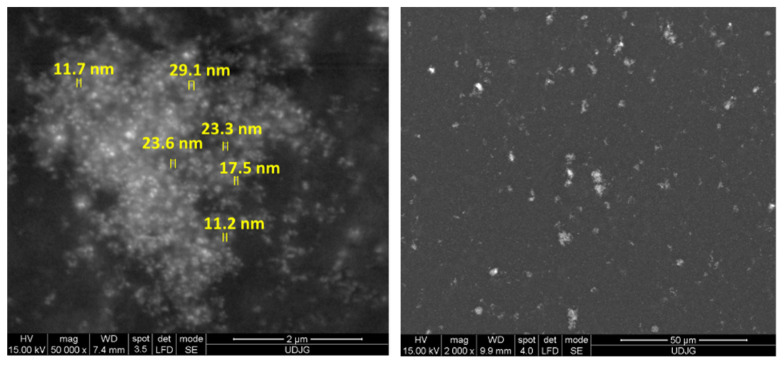
SEM micrographs of CeO_2_ nanoparticles.

At the beginning of the experiments, the dispersed phase of CeO_2_ was also characterized by SEM at different magnifications, as can be seen in Figure 1. In order to be analyzed with SEM, the CeO_2_ nanopowder was dispersed in absolute ethyl alcohol with a ratio of 1 mg of CeO_2_ nanoparticles to 30 mL of absolute p.a. ethyl alcohol. It remained dispersed in an ultrasonic bath for 3 min, after which it was pipetted onto a carbon strip and then visualized with SEM.

Figure 1 shows that the CeO_2_ nanoparticles were found to be in the form of agglomerations, but they retained their nanometric dimensions, which shows that the CeO_2_ nanoparticles with nanometric dimensions were used in this research work.

For the structural analysis of the pure Co and Co/nano-CeO_2_ layers that were obtained, the X-ray diffraction method was used, using Dron-3 equipment. XRD diffraction patterns were recorded using a cobalt anode (Co, λKa = 1.790300 Å) at a voltage of 30 KV and a current of 20 mA, in a range between 15 and 90°, and with a step of 0.05°/s, a time exposure of 3 s, and a total time/sample of 2 h and 13 min. The obtained spectra were analyzed using Match! 3 (http://www.crystalimpact.com/match, accessed on 21 May 2022) connected to the Crystallography Open Database.

The contact angle (AC) measurements with an accuracy of ±1° were made with OCA 15 EC, Dataphysics, Germany, connected to a laptop and controlled by SCA20 software, in the test syringe of which the Hank biological solution was inserted. The volume of the solution used for each measurement was 10 μL at a rate of 1 μL/s. For each sample, 5 measurements were taken from 5 different places on the surface of the sample, for a total of 100 records each, after which the average value was calculated.

For testing the surface roughness of the obtained layers, the portable roughness meter Surftest SJ-210 (Mitutoyo Corporation, Kanagawa, Japan) was used. The analysis length was 4 mm with a scan speed of 0.25 μm/s. A total of 5 measurements were taken for each sample, after which the average value was calculated.

## 3. Results

### 3.1. Layer Systems Obtained by Electrocodeposition

**Figure 2:**
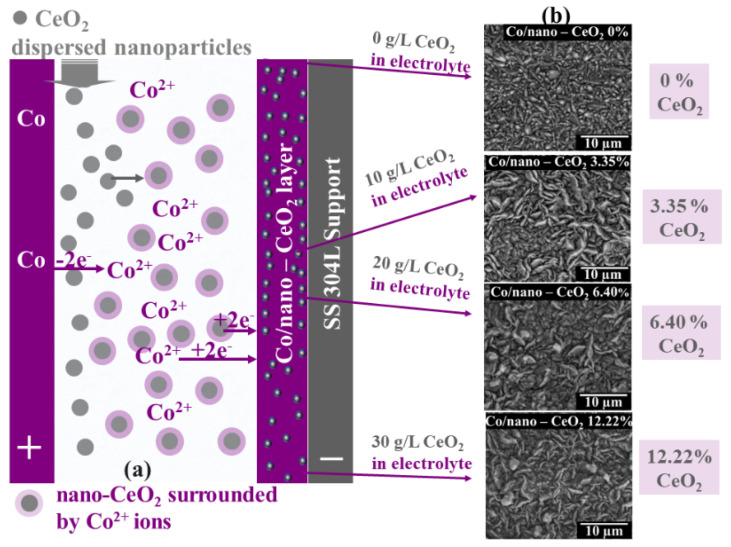
Schematic of nano-CeO_2_ electrocodeposition into cobalt matrix to obtain Co/nano-CeO_2_ nanostructured layers: (**a**) electrochemical cell with an SS 304L cathode on which the pure cobalt and nanostructured layers were electrodeposited, a cobalt sheet as the anode, a cobalt electrolyte with Co^2+^ ions, and dispersed nano-CeO_2_ particles, which were surrounded by ions from the solution after the immersion; (**b**) SEM micrographs of pure cobalt and Co/nano-CeO_2_ nanostructured layers obtained at different concentrations of dispersed CeO_2_ nanoparticles in the electrolyte, and having a different wt. percent of CeO_2_ embedded into the cobalt matrix.

The pure cobalt layer and nanostructured Co/nano-CeO_2_ layers were obtained using the electrocodeposition process by dispersing nano-CeO_2_ particles into a cobalt matrix using different concentrations of CeO_2_ in the cobalt plating electrolyte. The scheme of the procedure for obtaining the Co/nano-CeO_2_ layers is presented in Figure 2.

The systems that were obtained and studied in order to observe the effect of the degree of CeO_2_ nanoparticle inclusion in the cobalt matrix on the crystallite size of the matrix and on the hydrophobicity of the obtained nanostructured layers are presented in Table 2. The current density applied to the electrodeposition process affected the content of the embedded nano-CeO_2_ particles in the cobalt matrix [48]. The percentage incorporation of CeO_2_ nanoparticles in the Co matrix was determined by transforming the mass percentage of Ce (wt. %) from the general analysis obtained by SEM-EDX into the mass percentage of CeO_2_ using its molecular mass [48].

As can be seen in Table 2, the degree of inclusion of nanoparticles increased with an increase in the dispersed-phase concentration (CeO_2_); in the same system, the degree of nanoparticle inclusion decreased with increasing current density applied to the electrodeposition process. This effect was more accurately described in the published work [48].

### 3.2. Structural Characterization of Nanocomposite Layers by XRD

An X-ray diffraction (XRD) analysis was performed in order to identify the crystalline phases, as well as to estimate the crystalline size of the compounds formed on the studied pure Co surfaces compared to those on the Co/nano-CeO_2_ nanostructured layers.

**Figure 3:**
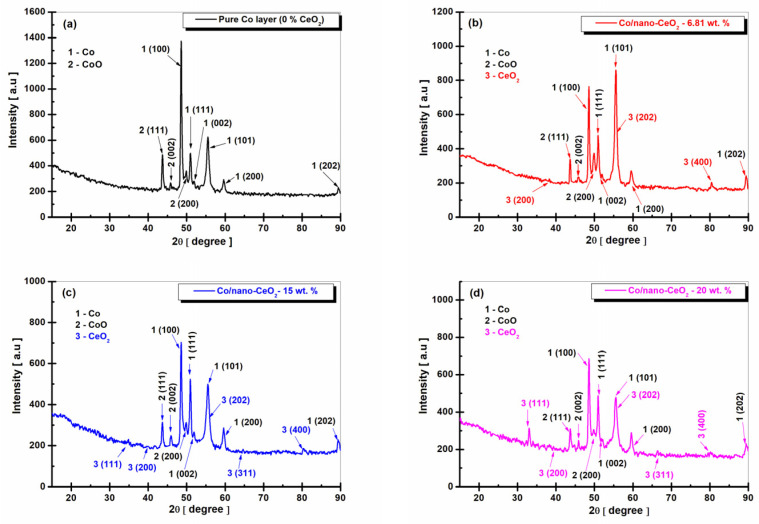
XRD patterns of electrodeposited layers obtained at a current density of 23 mA cm^−2^: (**a**) pure Co layer (0 wt. % CeO_2_), (**b**) Co/nano-CeO_2_—10 g/L layer (6.81 wt. % CeO_2_), (**c**) Co/nano-CeO_2_—20 g/L layer (15 wt. % CeO_2_), and (**d**) Co/nano-CeO_2_—30 g/L layer (20 wt. % CeO_2_).

**Figure 4:**
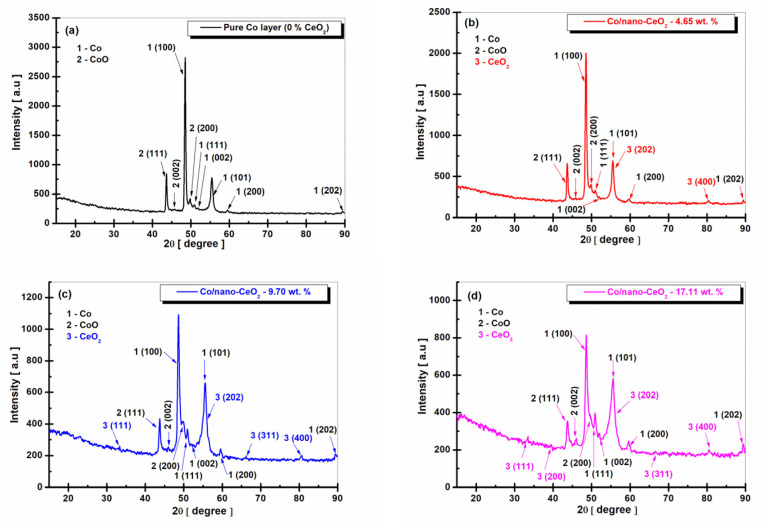
XRD patterns of electrodeposited layers obtained at a current density of 48 mA cm^−2^: (**a**) pure Co layer (0 wt. % CeO_2_), (**b**) Co/nano-CeO_2_—10 g/L layer (4.65 wt. % CeO_2_), (**c**) Co/nano-CeO_2_—20 g/L layer (9.70 wt. % CeO_2_), and (**d**) Co/nano-CeO_2_—30 g/L layer (17.11 wt. % CeO_2_).

**Figure 5:**
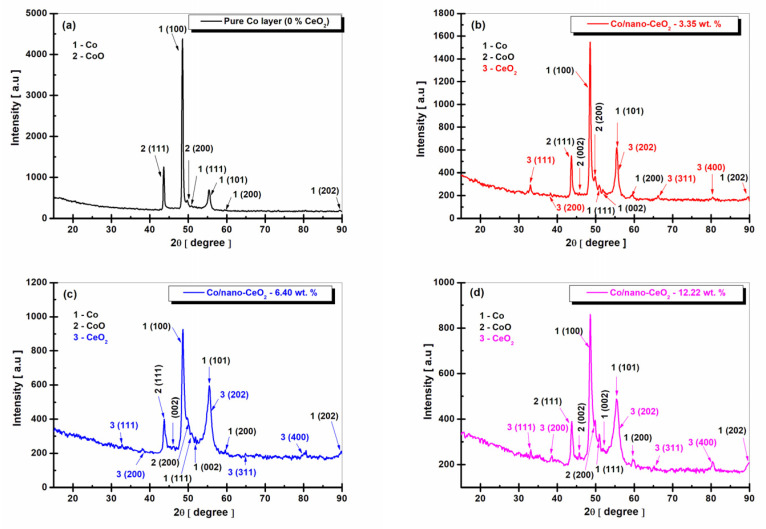
XRD patterns of electrodeposited layers obtained at a current density of 72 mA cm^−2^: (**a**) pure Co layer (0 wt. % CeO_2_), (**b**) Co/nano-CeO_2_—10 g/L layer (3.35 wt. % CeO_2_), (**c**) Co/nano-CeO_2_—20 g/L layer (6.40 wt. % CeO_2_), and (**d**) Co/nano-CeO_2_—30 g/L layer (12.22 wt. % CeO_2_).

Figure 3, Figure 4 and Figure 5 show the XRD diffraction patterns obtained at the current densities of 23, 48, and 72 mA cm^−2^ for the pure Co layers compared to the Co/nano-CeO_2_ nanostructured layer systems, obtained electrochemically by adding three concentrations of CeO_2_ nanoparticles (10 g/L, 20 g/L, and 30 g/L) into the cobalt plating bath.

At the concentrations of CeO_2_ used in the plating bath, the nanostructured layers embedded different concentrations of nano-CeO_2_ into the cobalt matrix, as can be seen in the following figures.

By following the XRD analysis using Match! 3, the following crystalline phases were identified for the pure Co electrodeposited layers. Figure 3a: Co-element at the diffraction angles (2θ) of 48.41° ± 0.4, 51.98° ± 0.12, 55.38° ± 0.32, corresponding to the crystallographic planes (Miller indices) of (100), (002), and (101), respectively. These detected phases were recorded in the database of the Crystallography Open Database (COD) program 96-901-1616, belonging to the hexagonal crystallization system, space group P63/mmc (194).

In addition, for the pure Co electrodeposited layers, the crystallographic planes of (111), (200), and (202), corresponding to the diffraction angles (2θ) of 50.95° ± 0.2, 59.82° ± 0.7, and 89.82° ± 0.9, respectively, were identified according to COD 96-901-2941 as belonging to the cubic crystallization system, space group Fm-3m (225). The crystalline phases of the CoO compound, identified by COD 96-153-3088 with the crystallographic planes of (111), (002), and (200), were also identified at diffraction angles (2θ) of 43.73° ± 0.34, 46.15° ± 0.23, and 49.85° ± 0.16, respectively, belonging to the cubic crystallization system, space group Fm-3m.

Similar results regarding the appearance of crystalline phases in pure cobalt layers have been reported in the literature by the author Vladimir Matveev [49], who studied the preferential orientation of cobalt nanoparticles using XRD analysis.

For the Co/nano-CeO_2_ (wt. 6.81, 15, and 20%) electrocodeposited nanocomposite layers, obtained at the current density of 23 mA cm^−2^, the peaks of the element Co and the CoO oxide were identified in similar positions as those of the pure Co electrodeposited layer with the characteristics and details mentioned above.

In addition to these peaks, the crystalline phases of the CeO_2_ nanoparticles appeared with the crystallographic planes of (111), (200), (202), (311), and (400), at 2θ diffraction angles of 33.57° ± 0.21, 39.28° ± 0.42, 55.90° ± 0.17, 66.55° ± 0.19, and 80.40° ± 0.13, respectively, identified with COD 96-434-3162 as part of the cubic crystallization system, space group Fm-3m.

From Figure 3, a decrease in the intensity of the Co peak can be observed with an increase in the dispersed-phase concentration, with the highest peak being present in the pure Co layer.

For the CeO_2_ phase shown in Figure 3b–d, there was an increase in the peak intensity according to the plane (111) with an increase in the dispersed-phase concentration in the cobalt matrix (from 6.81 wt. % to 20 wt. %), and a decrease in the peak intensity of CeO_2_ according to the plane (400) with an increase in the embedded CeO_2_ dispersed-phase concentration in the cobalt matrix (from 6.81 wt. % to 20 wt. %).

For each system studied, according to the applied current density, the average size of the crystals was also calculated using the Debye–Sherrer formula [50,51], as shown in Equation (1):

(1) $$d_{XRD}=\frac{0.9\lambda }{FWHM\cdot \cos\theta }$$

where *d_XRD_* is the crystal size; *λ* for Co K_α_ is 1.790300 Ǻ; FWHM (in radians) is the full width at the maximum half of the characteristic peak; and *θ* is the diffraction angle.

For the pure Co layer (0 wt. % CeO_2_) obtained at a current density of 23 mA cm^−2^ (Figure 3a), the average size of the Co crystals was 41.80 ± 0.6 nm, and for CoO it was 30.09 ± 0.5 nm.

For the Co/nano-CeO_2_ layer (6.8 wt. %) (Figure 3b) obtained at the same current density, the average crystal size was 33.80 ± 0.5 nm for the Co metal matrix, 15.27 nm for CoO, and 21.28 nm for CeO_2_. For the Co/nano-CeO_2_ (15 wt. %) layer (Figure 3c), the average crystal size was 30.07 nm for the Co metal matrix, 17.72 ± 0.4 nm for CoO, and 20.40 ± 0.5 nm for CeO_2_. In the case of the nanostructured layer with the addition of 30 g/L of nano-CeO_2_ (20 wt. %) (Figure 3d), the average crystal size was 25.80 ± 0.4 nm for the Co metal matrix, 23.71 ± 0.2 nm for CoO, and 17.41 ± 0.8 nm for CeO_2_. It was clearly shown that the nano-CeO_2_ particles embedded into the cobalt matrix caused a decrease in the size of the cobalt crystallites, from 41.80 ±0.6 nm for the pure electrodeposited cobalt to 25.80 ± 0.4 nm for the Co/nano-CeO_2_ nanostructured layer with 20 wt. % of embedded nanoparticles.

The size value of CeO_2_ included into the cobalt matrix was close to that given by the producing company for CeO_2_ nanoparticles as being <25 nm.

In Figure 4, the X-ray diffraction patterns of the electrodeposited layers, obtained at a current density of 48 mA cm^−2^, are presented.

From the XRD patterns of the Co/nano-CeO_2_ nanostructured layers obtained at a current density of 48 mA cm^−2^, as shown in Figure 4, peaks that were similar to those obtained at the current density of 23 mA cm^−2^ were observed. There was also a decrease in the intensity of the cobalt peak with an increase in the nano-CeO_2_ dispersed-phase concentration.

At the same time, several crystalline phases were identified at the corresponding peak of the Co element with the crystallographic plane (101) and a diffraction angle (2θ) of 55.38° ± 0.32, as identified with COD no. 96-901-1616. This is close to the crystalline phase of CeO_2_ identified by COD no. 96-434-3162, which had the crystallographic plane (202) and a diffraction angle (2θ) of 55.90° ± 0.17.

This phenomenon of the identification of two different phases relatively close together in the same peak is possible due to the increased heterogeneous germination in preferential crystallographic directions (Miller indices) [52,53].

These peaks of the cobalt matrix, which have several crystalline phases, were found only in the samples of nanostructured layers that included CeO_2_ nanoparticles in the cobalt matrix, thus being found in similar positions at all the studied current densities.

For the pure Co layer (0 wt. % CeO_2_) obtained at a current density of 48 mA cm^−2^ (Figure 4a), the average size of the Co crystals was 48.15 ± 0.9 nm, and for CoO it was 36.85 ± 0.4 nm.

For the Co/nano-CeO_2_—10 g/L layer (4.65 wt. % CeO_2_) obtained at the same current density, as shown in Figure 4b, the average crystallite size was 32.51 ± 0.8 nm for the Co metal matrix, 11.44 ± 0.2nm for CoO, and 23.32 ± 0.6 nm for CeO_2_. For the Co/nano-CeO_2_ 20 g/L layer (9.70 wt. % CeO_2_), as shown in Figure 4c, the average crystallite size was 29.79 ± 0.1 nm for the Co metal matrix, 14.51 ± 0.7 nm for CoO, and 16.02 ± 0.2 nm for CeO_2_. In the case of the nanostructured layer with the addition of 30 g/L of nano-CeO_2_ (17.11 wt. % CeO_2_), as shown in Figure 4d, the average crystal size was 22.87 ± 0.2 nm for the Co metal matrix, 15.96 ± 0.4 nm for CoO, and 17.14 ± 0.7 nm for CeO_2_.

In Figure 5, the X-ray diffraction patterns of the electrodeposited layers obtained at a current density of 72 mA cm^−2^ are presented.

Figure 5, which displays the XRD diffraction patterns corresponding to the layers obtained at the current density of 72 mA cm^−2^, shows peaks similar to those identified at the current densities of 23 mA cm^−2^ and 48 mA cm^−2^.

At a current density of 72 mA cm^−2^, for the pure Co layer (0 wt. % CeO_2_), the Co element had an average crystal size value of 54.65 ± 0.8 nm; for CoO, it was 39.79 ± 0.1 nm (Figure 5a).

When keeping the same current density for the Co/nano-CeO_2_—10 g/L layer (3.35 wt. % CeO_2_), as shown in Figure 5b, the average crystal size was 30.51 ± 0.1 nm for the Co metal matrix, 9.64 ± 0.4 nm for CoO, and 18.02 ± 0.6 nm for CeO_2_. For the Co/nano-CeO_2_ 20 g/L layer (6.40 wt. % CeO_2_), as shown in Figure 5c, the average crystal size was 28.17 ± 0.4 nm for the Co metal matrix, 9.16 ± 0.3 nm for CoO, and 17.26 ± 0.6 nm for CeO_2_. In the case of the nanocomposite layer with the addition of 30 g/L of nano-CeO_2_ (12.22 wt. % CeO_2_), as shown in Figure 5d, the average crystal size was 19.80 ± 0.5 nm for the Co metal matrix, 6.47 ± 0.1 nm for CoO, and 18.80 ± 0.3 nm for CeO_2_.

Other authors [54] have also observed a decrease in the size of the matrix crystallites with an increase in the dispersed-phase concentration in the plating electrolyte.

The explanation for the decrease in the crystallite size of the cobalt matrix when increasing the dispersed-phase concentration in the plating electrolyte is as follows. The electrocrystallization or electrodeposition process is governed by two competitive steps: (i) nucleation site formation and (ii) crystal growth [54]. More nucleation sites will result in the reduced growth of the matrix crystallites. By adding nano-CeO_2_ particles in the plating electrolyte, more nucleation sites are formed on the substrate, which prevents the lateral and vertical growth of the cobalt crystallites. As a result, the average crystallite size decreases when the nano-CeO_2_ concentration in the electrolyte increases [54].

Therefore, the inclusion of CeO_2_ nanoparticles in the cobalt matrix leads to a nanostructuring effect on the cobalt matrix by reducing the size of the cobalt crystallites, from 54.65 ± 0.8 nm in electrodeposited pure cobalt to 19.80 ± 0.5 nm in the Co/nanoCeO_2_ nanostructured layers. The smallest value of cobalt matrix crystallites was found for 12.22 wt. % of embedded nano-CeO_2_.

In the case of pure cobalt deposits, increasing the current density led to an increase in the size of the cobalt crystallites in the electrodeposited layer, from 41.80 ± 0.6 nm at 23 mA cm^−2^ to 54.65 ± 0.8 nm at highest current density of 72 mA cm^−2^. Meanwhile, for the nanostructured layers, the size of the cobalt matrix crystallites decreased when the current density was increased from 33.80 ± 0.5 nm at 23 mA cm^−2^ to 19.80 ± 0.5 nm at 72 mA cm^−2^.

The inclusion of CeO_2_ nanoparticles in the cobalt matrix also disrupted its preferential crystallization.

As can be seen from the XRD diffraction patterns, cobalt electrocrystallized preferentially according to the plane (100), which had the highest intensity. The peak corresponding to this plane of pure cobalt crystallization increased in intensity when the current density was increased (Figure 3a, Figure 4a and Figure 5a). The second crystallization plane of pure cobalt, (101), maintained approximately the same intensity at all current densities applied to the electrodeposition of pure cobalt.

In the case of the nanostructured layers, this preferential crystallization according to the plane (100) of the cobalt in the metallic matrix decreased, and the corresponding peak decreased in intensity with increasing CeO_2_ dispersed-phase concentration (Figure 3b–d, Figure 4b–d and Figure 5b–d). Instead, there was an increase in the intensity of the peak corresponding to the crystallization plane (101).

### 3.3. Nanostructuring Effect of Nano-CeO_2_ Particles Embedded into the Cobalt Matrix

The size of a crystallite generally corresponds to the coherent volume of material for the respective diffraction peak. Sometimes, it also corresponds to the size of the grains in a powder sample or the thickness of the thin polycrystalline film or bulk material. The Scherrer equation was used to determine the particle size of powdered crystals, as shown in Equation (1).

The analysis of the X-ray diffraction (XRD) diagrams, constructed for the pure cobalt layers as well as for the Co/nano-CeO_2_ nanostructured layers, allowed for the evaluation of the nanostructuring effect of CeO_2_ nanoparticles by incorporating them into the cobalt matrix through electrolytic codeposition.

**Figure 6:**
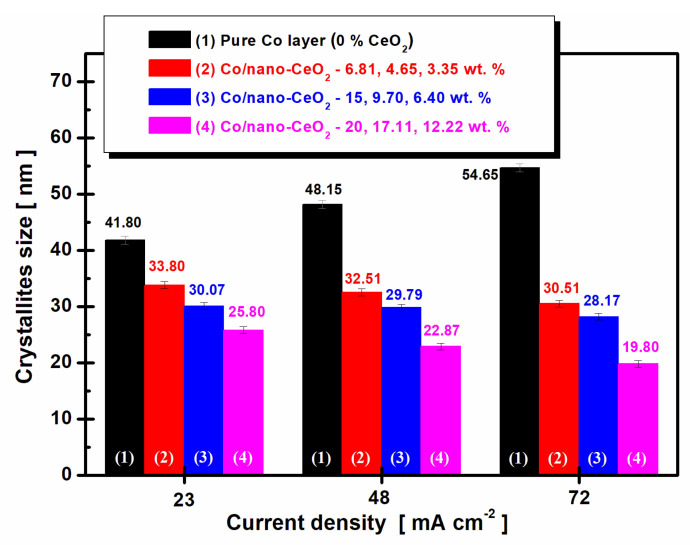
The nanostructuring effect of CeO_2_ nanoparticles embedded in the cobalt matrix by the electrocodeposition process.

The effect of the inclusion of nano-CeO_2_ into the cobalt matrix through the electrocodeposition process on the size of the cobalt crystallites is shown in Figure 6.

As can be seen in Figure 6, an increase in the current density caused an increase in the size of cobalt crystallites in the case of the electrodeposition of pure cobalt. Thus, the size of cobalt crystallites increased from 41.80 ± 0.6 nm to 54.65 ± 0.8 nm.

By adding cerium oxide nanoparticles, CeO_2_, (Figure 6), a decrease in cobalt matrix crystallites was induced with increasing current density; therefore, the nanoparticles had a nanostructuring effect on it. This nanostructuring effect of cobalt matrix crystallites was more pronounced as the dispersed-phase concentration added to the cobalt electrolyte increased.

Thus, at the highest concentration of 30 g/L of CeO_2_ nanoparticles added into the cobalt electrolyte, the cobalt nanocrystallites had the smallest dimensions, from 25.80 ± 0.4 nm to 19.80 ± 0.5 nm, and they approached the size of CeO_2_ nanoparticles (<25 nm given by the manufacturer).

The particle size of the CeO_2_ nanoparticles, calculated from the XRD diffraction patterns of the electrodeposited nanocomposite layers, was between 16.02 ±0.2 nm and 23.22 ±0.6 nm, which was very close to the average size of <25 nm given by the manufacturer.

As can be seen from Figure 3, Figure 4 and Figure 5 on the electrodeposited pure cobalt layers, cobalt oxide (CoO) appeared with a significant diffraction peak in the XRD diffraction pattern diagram. This proved that the surface of the electrodeposited pure cobalt oxidized in the presence of oxygen in the air, predominantly forming a passive CoO film.

Also among the compounds identified in the Co/nano-CeO_2_ nanocomposite layers were cobalt oxides, with the most important being CoO, which confirmed that the cobalt matrix in the nanocomposite layers oxidized similarly to the pure cobalt layer.

### 3.4. Surface Wettability and Roughness Characterization

In addition to the morphological, topographical, and structural characterization of the obtained surfaces, the biological response was also examined. Biocompatibility, which refers to the ability of a material to interact with human tissue, is of great importance.

To detect the biocompatibility of the obtained surfaces, a wetting test was performed using contact angle (CA) measurements. Moisture is one of the main physical factors influencing the ability of a liquid phase to maintain contact with the surface of a solid phase.

This phenomenon is mainly based on the intermolecular interaction of the liquid–solid phase [55].

In this paper, the osseointegration of the obtained surfaces—that is, the ability of the surfaces to interact favorably with the biological environment—was investigated. This property is expressed by the angle of contact with a fluid, ranging from 0° on highly hydrophilic surfaces to more than 90° on hydrophobic surfaces. This biological response of implanted biomaterials is usually affected by surface wetting, which affects protein adsorption, platelet adhesion, and blood clotting. Hydrophilic surfaces maintain the conformation and function of proteins, while hydrophobic textures trigger protein denaturation by exerting conformational changes [56].

The ability of cells to attach and migrate to an implant surface is determined by protein adsorption. Hydrophilic surfaces have a higher affinity for proteins than hydrophobic surfaces. In addition, it has been shown that the surfaces of biomaterials used as implants that have a high degree of hydrophilicity promote the differentiation and maturation of osteoblasts, thus contributing to the acceleration of bone integration [57,58].

**Figure 7:**
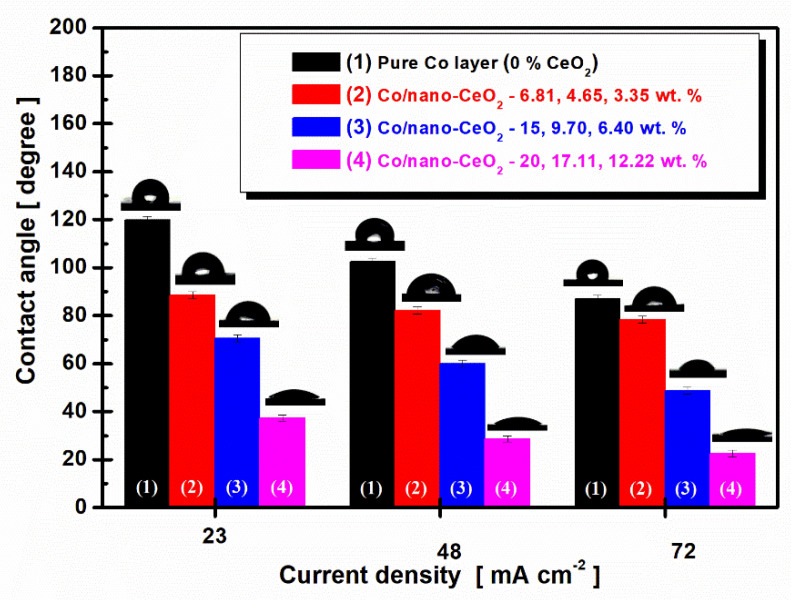
Variation of contact angle according to current density at deposition times of 90 min for: (**1**) pure Co layer (0% CeO_2_), (**2**) Co/nano-CeO_2_—10 g/L layer (6.81, 4.65, and 3.35 wt. %), (**3**) Co/nano-CeO_2_—20 g/L layer (15, 9.70, and 6.40 wt. %), and (**4**) Co/nano-CeO_2_—30 g/L layer (20, 17.11, and 12.22 wt. %).

The average values of the measurements on different layers depending on the current density are shown in Figure 7.

From Figure 7, we can observe that the average values of the contact angle decreased with an increase in the current density and an increase in the nanoparticle concentration. For the pure Co layer (0% CeO_2_) at a current density of 23 mA cm^−2^, we found a contact angle value of 111.92° ± 0.64. At the same current density, we noticed that this value for the contact angle decreased with an increase in the concentration of CeO_2_ nanoparticles in the cobalt electrolyte at 88.46° ± 0.51 for Co/nano-CeO_2_—10 g/L (6.81 wt. %), at 70.52° ± 0.43 for Co/nano-CeO_2_—20 g/L (15 wt. %), and at 37.39° ± 0.75 for Co/nano-CeO_2_—30 g/L (20 wt. %).

At a current density of 42 mA cm^−2^ for the pure Co layers (0% CeO_2_), the value of the contact angle decreased at 102.46° ± 0.35. For the surface with 10 g/L of CeO_2_ (4.65 wt. %), the contact angle value was 82.27° ± 0.82; for the surface obtained with 20 g/L of CeO_2_ (9.70 wt. %), the value was 59.99° ± 0.54; and a value of 28.65° ± 0.49 was reached for the surface obtained with the addition of 30 g/L of CeO_2_ (17.11 wt. %). At a current density of 72 mA cm^−2^ for the pure Co layers (0% CeO_2_), the value of the contact angle decreased to 96.98° ± 0.68. For the surface with an addition in an electroplating bath of 10g/L of CeO_2_ (3.35 wt. % CeO_2_), the contact angle value was 78.41° ± 0.78. For the surface obtained with the addition of 20 g/L of CeO_2_ (6.40 wt. % CeO_2_), a value of 48.69° ± 0.61 was reached. For the surface obtained with 30 g/L of CeO_2_ (12.22 wt. % CeO_2_), the value of the contact angle was 22.59° ± 0.61. This trend of a decrease in the contact angle with increasing current density has also been reported in the literature by other authors [55]. It is known that roughness is a key parameter that directly influences the wettability of surfaces [54].

In Table 3, the average value of the roughness measurements (R_a_) at different current densities are presented for the pure electrodeposited cobalt and Co/nano-CeO_2_ (10, 20, and 30 g/L) nanocomposite layers.

From Table 3, it can be observed that a decrease in the values of the contact angle was consistent with an increase in the values obtained when studying the roughness, as supported by the Wenzel equation (Equation (2)) [59,60]:

(2) $$\cos\theta_{W}=r\cos\theta_{Y}$$

where *r* is the average roughness, *θ_W_* is the measured contact angle value, and *θ_Y_* is the Young contact angle value for an ideal smooth surface [59,60].

This equation shows that increased roughness values reduce the contact angle, which means a more hydrophilic surface [59,60].

### 3.5. Correlation between Crystallite Size Dimensions of Nanocomposite Layers and Wettability

As has been shown in previous results, the size dimensions of cobalt crystallites are affected by the electrocodeposition of nano-CeO_2_ particles, as the dimension values decreased with an increase in the applied current density during the electrocodeposition process. The surface hydrophobicity of all layers obtained by electrodeposition was affected by the current density applied during the electrodeposition process.

**Figure 8:**
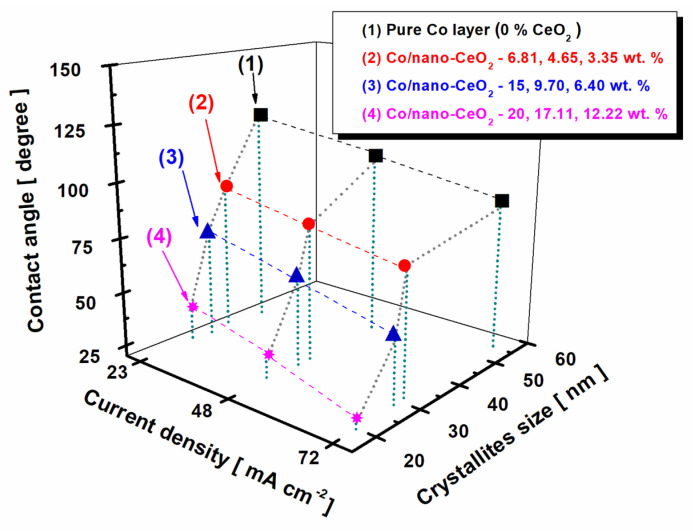
Correlation between nanostructuring effects of nano-CeO_2_ embedded into a cobalt matrix and the contact angle of resulting nanostructured layers.

In Figure 8, the correlation between the nanostructuring effects of nano-CeO_2_ embedded into a cobalt matrix and the contact angle of the resulting nanostructured layers is presented.

From Figure 8, it can be observed that the dimensions of the cobalt crystallites affected the wettability of the nanostructured layers. A decrease in crystallite size caused a decrease in the contact angle value of the nanostructured layers. The effect was similar with an increase in the current density and increases in the nanoparticle concentration.

For the pure Co layer (0% CeO_2_) obtained at a current density of 23 mA cm^−2^, we found a contact angle value of 111.92° ± 0.64 and a Co crystallite size of 41.80 nm ± 0.6. At the same current density, it can be seen that this contact angle value decreased with a decrease in the cobalt crystallite size, and also with an increase in the concentration of CeO_2_ nanoparticles embedded into the cobalt matrix. The contact angle value decreased to 88.46° ± 0.51 with a crystallite size of 33.80 nm ± 0.5 for Co/nano-CeO_2_—10 g/L (6.81 wt. %). For Co/nano-CeO_2_—20 g/L (15 wt. %), with a cobalt crystallite size of 30.07 nm ± 0.1, the value of the contact angle decreased to 70.52° ± 0.43. For the nanostructured layer of Co/nano-CeO_2_—30 g/L (20 wt. %), with a crystallite size of 25.80 nm ± 0.4, the value of the contact angle decreased to 37.39° ± 0.75.

At an applied current density of 48 mA cm^−2^ for the pure Co layers (0% CeO_2_), with a crystallite size of 48.15 nm ± 0.9, the value of the contact angle decreased to 102.46° ± 0.35. For the nanostructured surface with 10 g/L of CeO_2_ (4.65 wt. %), the contact angle value was 82.27° ± 0.82 and the crystallite size had dimensions of 32.51 nm ± 0.8. For the surface obtained with 20 g/L of CeO_2_ (9.70 wt. %), the cobalt crystallite size was 29.79 nm ± 0.1 and the value of the contact angle was 59.99° ± 0.54. The contact angle reached a value of 28.65° ± 0.49 for the nanostructured surface obtained with the addition of 30 g/L of CeO_2_ (17.11 wt. %), with a cobalt matrix crystallite size of 22.87 nm ± 0.2.

By further increasing the applied current density to 72 mA cm^−2^ for the pure Co layers (0% CeO_2_), the pure cobalt crystallite size increased to 54.65 nm ± 0.8 and the value of the contact angle decreased to 96.98° ± 0.68. For the surface with an addition in an electroplating bath of 10g/L of CeO_2_ (3.35 wt. % CeO_2_), the cobalt matrix crystallite size was 30.51 nm ± 0.1 and the contact angle value was 78.41° ± 0.78. For the nanostructured surface obtained with the addition of 20 g/L of CeO_2_ (6.40 wt. % CeO_2_), the cobalt matrix crystallite size was 28.17 nm ± 0.4 and the contact angle reached a value of 48.69° ± 0.61. For the nanostructured surface obtained with 30 g/L of CeO_2_ (12.22 wt. % CeO_2_), the cobalt matrix crystallite size was 19.80 nm ± 0.5 and the value of the contact angle decreased to 22.59° ± 0.61.

## 4. Conclusions

This research work presents the effect of the nanostructuring of CeO_2_ nanoparticles incorporated into a cobalt matrix during the electrocodeposition process and the correlation between the nanostructuring and the wetting properties of the obtained nanostructured layers at different current densities.

The analyses of the contact angle showed that when the concentration of nanoparticles, the roughness, and the current density were increased, the surfaces became more hydrophilic, indicating that from a biological point of view, this behavior led to a better osseointegration of the possible implant.

From X-ray diffraction patterns, it was clear that by increasing the current density in the case of pure cobalt layers, an increase in the size of the cobalt crystallite occurred in the electrodeposited layer, from 41.80 ± 0.6 nm to 54.65 ± 0.8 nm.

For the nanostructured layers, the crystallite size decreased with increasing current density, which confirmed the nanostructuring effect of nano-CeO_2_ particles codeposited with cobalt, from 54.65 ± 0.8 nm in the electrodeposited pure cobalt to 19.80 ± 0.5 nm in the Co/nanoCeO_2_ nanostructured layers. The smallest value for the cobalt matrix crystallites resulted from 12.22 wt. % of embedded nano-CeO_2_.

It was also observed from the XRD analysis that the CeO_2_ nanoparticles incorporated into the Co matrix disturbed the preferential orientation of the cobalt crystallites from the cobalt matrix, while at the same time decreasing the intensity of the predominant peak corresponding to the pure Co layer.

The inclusion of CeO_2_ nanoparticles into the cobalt matrix led to the nanostructuring of the cobalt matrix by reducing the size of the cobalt crystallites.

A correlation was shown between the contact angle and the crystallite size of the cobalt matrix, as a decrease in the values of the contact angle was observed with a decrease in the size of the crystallites, which in turn decreased with increasing current density, dispersed-phase concentration, and dispersed-phase embedding into the cobalt matrix.

## Figures and Tables

**Table 1:** Physical and chemical characteristics of cerium oxide.

Characteristics	Technical Specifications
Molecular mass	172.11 g/mol
Color	White-pale, yellow pal
Morphology	Nanopowder
The specific size of the surface	35–55 m^2^/g
Particle size	<25 nm
Density at 20 °C	7.22 g/cm^2^
Melting point	2340 °C
Boiling point	3500 °C

**Table 2:** The studied layer systems and the dispersed phase concentrations present in the obtained nanostructured layers [48].

Amount of Dispersed Nano-CeO_2_ Added to Plating Electrolyte	Current Density	Amount of Dispersed Nano-CeO_2_ Added to Plating Electrolyte
0 g/L CeO_2_	23	0
	48	0
	72	0
10 g/L CeO_2_	23	6.81 ± 0.2
	48	4.65 ± 0.3
	72	3.35 ± 0.1
20 g/L CeO_2_	23	15.0 ± 0.4
	48	9.70 ± 0.1
	72	6.40 ± 0.2
30 g/L CeO_2_	23	20.0 ± 0.1
	48	17.11 ± 0.2
	72	12.22 ± 0.3

**Table 3:** Average value of roughness measurements (R_a_) for layers obtained at different current densities.

Surface Studied	Current Density [mA/cm^2^]	R_a_ [µm]
Pure Co layer (0% CeO_2_)	23	0.142 ± 0.002
	48	0.207 ± 0.004
	72	0.297 ± 0.001
Co/nano-CeO_2_—10 g/L layer (6.81, 4.65, and 3.35 wt. %)	23	0.346 ± 0.006
	48	0.421 ± 0.003
	72	0.504 ± 0.005
Co/nano-CeO_2_—20 g/L layer (15, 9.70, and 6.40 wt. %)	23	0.609 ± 0.001
	48	0.693 ± 0.003
	72	0.755 ± 0.009
Co/nano-CeO_2_—30 g/L layer (20, 17.11, and 12.22 wt. %)	23	0.849 ± 0.004
	48	0.991 ± 0.007
	72	1.233 ± 0.003

## Data Availability

The data presented in this study are available on request from the corresponding author.
